# What contributes to good patient outcomes in the home treatment of the severely mentally ill: study protocol of a multi-centre analysis

**DOI:** 10.1186/1471-244X-13-283

**Published:** 2013-11-05

**Authors:** Constance Stegbauer, Katja Goetz, Erik Bauer, Beate Bestmann, Thomas Ruprecht, Joachim Szecsenyi, Anke Bramesfeld

**Affiliations:** 1 AQUA – Institute for Applied Quality Improvement and Research in Health Care, Maschmühlenweg 8-10, 37073 Göttingen, Germany; 2 Department of General Practice and Health Services Research, University Hospital Heidelberg, Voßstr. 2, 69115 Heidelberg, Germany; 3 Scientific Institute of TK for Benefit and Efficiency in Health Care (WINEG), Bramfelder Str. 140, 22305 Hamburg, Germany; 4 Techniker Krankenkasse, Bramfelder Str. 140, 22305 Hamburg, Germany; 5 Department Epidemiology, Social Medicine and Health System Research, Hannover Medical School, Carl-Neuberg-Str. 1, 30625 Hannover, Germany

**Keywords:** Quality assessment, Mental health services, Integrated care, Claims data, Health services research

## Abstract

**Background:**

While evidence is available that home treatment could be effective for treating severe mental illness, there is a lack of evidence on what exactly makes home treatment effective. The study presented here aims to develop recommendations for structures and processes in home treatment that are necessary for its effectiveness.

**Methods/Design:**

14 provider networks of home treatment for severe mental illness will be analyzed and compared according to their structures, processes and patient-related outcomes. Data will be drawn from health care claims data, routine assessments of psychosocial functioning, and from questionnaires on structures and processes. The primary outcome will be psychosocial functioning; secondary outcomes, quality of life and days spent in hospital. The relation between structures and processes on one hand side and outcomes on the other side will be identified by multilevel analysis. In addition, focus groups with patients, relatives and network staff will be held to add further insight into relevant processes. All networks will receive individual quality reports, providing them with feedback on the results of this research and benchmarking them against the average. Based on this research, recommendations for processes and structures of home treatment will be developed.

**Discussion:**

The research will use longitudinal data on outcomes routinely assessed since 2009 and claims data. Routine data is also used for the assessment of structures and processes. By way of additional questionnaires developed in discussion with providers, further relevant factors can be included. The approach of this study becomes more comprehensive by conducting focus groups with patients, relatives and providers and by having the chance to evaluate the results with the networks by providing feedback of results. Several factors such as outcomes related to regional availability of hospital beds or size of networks might limit this study.

## Background

There is some international evidence available that home treatment of severely mentally ill persons provided by multidisciplinary psychosocial intervention teams has the potential to be effective. Home treatment was shown to reduce the need for inpatient treatment; it decreased suicidality, improved patients’ functional status and also increased satisfaction with treatment [1-3]. The efficiency of home treatment as part of integrated care models has also been shown under the care conditions of the German health care system [4]. While models of home treatment are currently becoming more and more popular in Germany, as well as in other Western countries, it is not known what structures and processes are needed to make home treatment of the severely mentally ill effective with regard to patient outcome. However, it is known that the efficiency of home treatment is closely related to its structures and processes. The research described in this article aims to identify structures and processes in home treatment of severely mentally ill persons that can be linked to patient-related outcomes. The research question to be answered is therefore:

“What structures and processes are necessary for providing effective and needs-related home treatment to severely mentally ill persons within the context of the German health care system?”

The outcome of this research should provide the basis for evidence-based recommendations on how structures and processes should be in the home treatment of severely mentally ill persons.

## Methods/Design

The research question will be addressed by analyzing 14 (13 + 1) regional care networks that provide similar, but nonetheless individually different home treatments within the framework of integrated care.

### The networks/samples

In 2009, one of the largest statutory health insurance companies in Germany, the Techniker Krankenkasse (TK) (8.5 million insured persons), set up a model for the integrated care of severely mentally ill persons called "NetzWerk psychische Gesundheit" (NWpG). Today, this model is provided by networks in a number of regions all over Germany. The networks aim to improve the coordination and continuity of care, thus reducing the need for inpatient care. The core services that all networks mandatorily offer include: home treatment and case-management, socio-therapy, psychoeducation, 24-hour crisis-hotline as well as the possibility for patients to stay temporarily in a crisis intervention apartment instead of hospitalization. The networks receive an annual fixed budget per patient to organize their care based on the patient’s individual needs and preferences. Beyond these core elements, the networks are free to choose how they organize themselves and with what other services they cooperate. This has led to differences in how regional networks are run. There are networks whose organization centres around office-based psychiatrists who closely collaborate with ambulatory nursing services to provide home treatment. Other networks are built on the structure of existing providers of psychosocial care where social workers or nurses might provide home treatment. Psychiatrists are also involved in these models but play a less prominent role in service provision and in particular, in navigating patients through the services. Some networks collaborate more closely with vocational rehabilitation or housing facilities; others offer services also for children. One of the networks distinguishes itself from the other networks (13) that focus on severe mental illness by specializing in eating disorders (+1).

Only patients insured with specific statutory insurance companies, for example the TK, are eligible for the services provided by the 13 + 1 networks. Patients are recruited by the TK based on a specific predictive risk model that uses claims data on diagnosis, medication and past hospitalization [5]. Patients who consent to participate in the service model are enrolled in the networks. Since 2009, more than 6000 patients have been recruited and treated.

**Figure 1:**
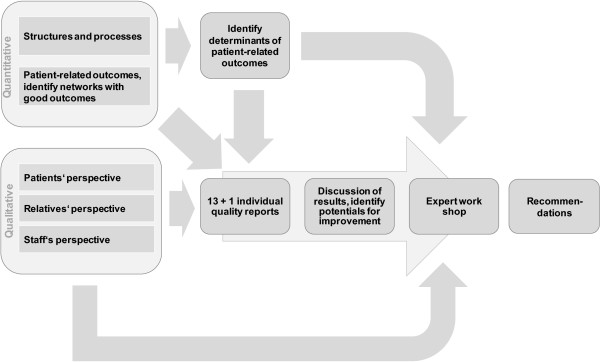
Flow and contents of analyzing 13 + 1 networks providing home treatment for the severely mentally ill.

A very comprehensive dataset is needed in order to analyze these networks and to be able to determine what structures and processes promote the provision of effective and needs-related treatment. Therefore, the study comprises of three methodological approaches (triangulation): a quantitative approach, a qualitative approach and a synthesis and feedback approach (see also Figure 1).

#### ***Quantitative approach***

The quantitative approach aims to assess in detail care structures, processes and patient-related outcomes. It will look for differences in the 14 networks in respect to these measures. Structures and processes will be related to outcomes by multilevel regression analysis. The following questions will guide the quantitative research:

1. How do the 13 + 1 networks differ in respect to structures and processes?

2. How do the 13 + 1 networks differ in respect to their patient-related outcomes?

3. What structures and processes are related to patient-related outcomes?

Structures to be assessed could include, for example, professional composition of the network, availability of supervision and number of patients per case manager. Processes to be assessed could include number of patients receiving home treatment, number of case review meetings per patient, time case managers spend with patients and additional services used by patients. Data on structures and processes will be drawn from four different sources: (1) claims data of the health insurance companies that include data on service use, medication and diagnosis; (2) annual reports from the networks to the health insurance companies; (3) additional assessment of structures and processes by way of a questionnaire that will be answered by the management of the networks; and by (4) a questionnaire aimed at the staff of the networks.

Both questionnaires (the questionnaire to be answered by network-management and the one for the staff) will be compiled, based on a systematic literature review of topics relevant to the structures and processes, i.e., how the networks function. These topics will be presented to the networks. Each network will be visited and the topics will be discussed in detail with the network’s management and staff. These discussions will focus on whether management and staff believe that the topics compiled are relevant, those which are missing, and whether it is feasible to obtain data on these topics.

Based on this information, the final questionnaires will be compiled, sent for comments to the networks and pre-tested.

Patient-related outcomes of a network are primarily defined as the average improvement in psychosocial functioning that network-enrolled patients achieve. Further, the improvement of a patient’s quality of life as well as the average reduction in the number of hospital admissions and days spent in hospital will be used as secondary outcomes of a network.

Data on the patients’ psychosocial functioning and on the quality of life are available by routine assessment within the networks. After enrollment and consecutively, every six months, case managers routinely assess psychosocial functioning by means of the Health of the Nation Outcome Scale (HoNOS). Patients assess their quality of life by means of the WHO-Quality of Life (WHOQoL). Data on these outcomes have been collected for all patients insured by the TK from 2009 up until now.

Data on hospitalization is available through the claims data of the TK. Patient-reported outcomes (HoNOS and WHOQol) will be linked to the claims data by pseudonyms. In this way, the aggregate and average outcomes for each network can be determined.

By way of multilevel regression, primary and secondary outcomes will be set in relation to structures and processes.

#### ***Qualitative approach***

The qualitative approach focuses on expectations, hopes and needs of the patients, their relatives and network staff. It aims to add to the results of the quantitative approach by offering explanations and additional material for understanding and interpreting the data. Therefore, focus groups with patients, patients’ relatives and providers will be held in four of the networks and in the network that specializes in eating disorders. The following questions will guide the focus group discussions:

1. What expectations, needs and hopes do patients have concerning service provision and cooperation within provider networks? What kind of processes help to meet these expectations, needs and hopes?

2. What expectations, needs and hopes do relatives have concerning service provision and cooperation within provider networks? What kind of processes help to meet these expectations, needs and hopes?

3. What expectations, needs and hopes do providers have concerning service provision and cooperation within provider networks? What kind of processes help to meet these expectations, needs and hopes?

Networks will be selected for focus group participation according to their differences in structures and processes or patient-related outcomes. Selected networks will assist the project in recruiting participants. The discussion in the focus groups will be audio recorded and then analyzed using the content analysis methodology.

#### ***Synthesis and feedback***

In this part of the research results of both the quantitative and qualitative approach will be merged and results fed back to the networks. Each participating network will receive a customized feedback report that presents its own quantitative results on structures, processes and outcomes in relation to the benchmark of the average results of all networks. This will be a specific part of the feedback report and, hence, different for each network. In addition, these individual reports will also contain a general part where overall results of the quantitative analysis will be presented, in particular those concerning structures and processes that determine a good outcome. Further, in the general part of the report, the outcomes of the focus group discussions will be presented to all of the networks. In a next step, it is intended that all networks be visited by the project team to present, explain and discuss the results, in particular the individual results with the network management and staff. This should be the basis on which networks develop their own targets for the improvement of structures and processes.

This methodology of providing individual benchmarked feedback to services, discussing them and, on this basis, developing targets for improvement, is derived from the experience and evidence that the AQUA-Institute, which is the lead of this study, has gathered while conducting the European Practice Assessment (EPA) [6]. EPA is based exactly on this method and as such, the project will be able to benefit from prior existing infrastructure such as the Visotool®, an internet-based feedback tool to visualize results.

The experiences gained from the discussion of the feedback reports together with the overall results from the quantitative and qualitative research approaches will be used to form recommendations for necessary structures and processes in networks providing home treatment to severely mentally ill persons in Germany. These recommendations will be presented, discussed and finalized in a workshop with all relevant stakeholders at national level. This would include representatives of the networks, patients and their families, medical and social service providers, health insurance companies, policy makers and researchers.

### Ethics and data protection

Ethical approval for the quantitative approach has been obtained from the ethics committee at the State Medical Chamber of Lower Saxony. They voted that no formal approval for the quantitative approach is required since no direct patient contact is involved. Information on patients’ characteristics, patient-related outcomes, patient service utilization and other process information will be conducted from pseudonymized claims and primary data. Patient-related results will only be published in an aggregate form and at network level. Ethical approval for the qualitative approach will be obtained from the University of Heidelberg in the context of preparation of the focus groups which will start the following year.

## Discussion

More and more care models are being implemented in Germany as well as worldwide that offer in one way or another home treatment, case management and multi-disciplinary care to persons who are severely mentally ill [7]. While there is evidence that such approaches to treatment can be effective [4,8], there has not been much research yet as to what elements of these services contribute to their effectiveness. What the sparse research on this issue does reveal is that the following aspects do indeed contribute to the effectiveness of home treatment: visiting patients regularly at home and having dual responsibility for both health and social care [9]. The relevance of the number of patients per case manager [10] and the intensity of care [8] are discussed controversially with regard to their impact on patient outcomes.

By way of this study, we aim to add evidence to the discussion on what are the relevant structures and processes for successful home treatment of severely mentally ill persons.

One of the strengths of this research will be that it incorporates a comprehensive approach, including quantitative and qualitative methods and a process of feedback of results to services. This feedback process will enable the validation of results in relation to the day-to-day practice of services. In addition, the feedback process provides the study subjects, e.g. the networks, with benchmark data on their structures and processes which could provide a basis for service improvement. A further strength of the research relates to the use of various longitudinal data sources for determining patient-related outcomes (claims data and routine assessment of psychosocial functioning and quality of life). Further, we will also be able to draw on various data sources for determining networks structures and processes, including questionnaires developed with the staff of the networks. By developing these questionnaires in an intensive discussion and exchange process with the networks’ management and staff, we hope to be able to consider the most relevant factors for the quality of services from the provider’s point of view.

One limitation of this research is related to the fact that while network outcomes will be determined by longitudinal data that can date back to 2009, all structures and processes will be assessed using current cross-cutting analysis. So it might be the case that patient improvement achieved in 2010 was accomplished with a slightly different service structure. We need to consider this when analyzing and interpreting the data.

Further risks and challenges of this research are related to the following parts of the study:

• Differences in network outcomes might be related also to service provision, as well as to patient characteristics, in particular their medical risk profiles. We will have to take this into account when using information from the claims data. Since all patients in this study were selected by the TK using predictive modelling, all patients should have comparable risk-profiles [11]. We will be able to attain patients’ risk profiles from the TK.

• Further differences in outcomes, represented by the average changes in inpatient service use, might be related more to the regional availability of hospital beds than to treatment factors. There are reports that in particular in regions where there is an excess supply of hospital beds, in particular, beds in psychosomatic-psychotherapeutic units, it becomes difficult to keep patients out of hospital, despite providing home treatment. We will have to consider this when interpreting the results. Therefore, close dialogue with the networks to understand context factors is necessary throughout this research.

• A final risk could be that we might not find any differences between networks with regard to patient-related outcomes. This could be caused by HoNOS and WHOQoL not being care sensitive to the patient group of the home treatment networks. It might also be that simply all networks are performing equally well on the outcome side. However, besides the primary outcome in psychosocial functioning, we have several secondary outcomes including service use. Further, in the event that all networks are performing equally well on outcomes, despite different structures and processes, the results from the focus groups will become even more relevant to explain results and determine priorities in structures and processes.

## Conclusion

In light of the rising popularity of home treatment for severely mentally ill persons, it is necessary and timely to add evidence to the discussion of what structures and processes are needed – or better still – what structures and processes would be essential for well-functioning home treatment that supports patients’ recovery and addresses their needs best. This research aims to contribute to this.

## Competing interests

TR is employed by the TK which is the health insurance company that insures the patients in the networks under evaluation. He implemented NWpG and served as project manager from 2007 to 2011. BB is employed by WINEG which is a research institute belonging to the TK. However, WINEG is independent from the operating business of the TK and reports solely to its steering board. Other authors declare that they have no competing interests.

## Authors’ contributions

CS wrote the paper, AB supervised her, AB, KG, BB, JS developed the study design, TR provided information about and contacts to networks, EB is accountable for data preparation and statistical analysis. All authors have read and commented on the paper. All authors read and approved the final manuscript.

## Pre-publication history

The pre-publication history for this paper can be accessed here:

http://www.biomedcentral.com/1471-244X/13/283/prepub
